# Barriers to lifestyle changes for prevention of cardiovascular disease – a survey among 40–60-year old Danes

**DOI:** 10.1186/s12872-017-0677-0

**Published:** 2017-09-12

**Authors:** Jesper Bo Nielsen, Anja Leppin, Dort e Gyrd-Hansen, Dorte Ejg Jarbøl, Jens Søndergaard, Pia Veldt Larsen

**Affiliations:** 1 Research Unit for General Practice, Department of Public Health, University of Southern Denmark, J.B.Winsløwvej 9, DK-5000 Odense, Denmark; 2 Unit for Health Promotion Research, Department of Public Health, University of Southern Denmark, Niels Bohrs Vej 9, DK-6700 Esbjerg, Denmark; 3 COHERE, Department of Public Health, University of Southern Denmark, J.B.Winsløwvej 9, DK-5000 Odense, Denmark; 4 Epidemiology, Biostatistics and Biodemography, Department of Public Health, University of Southern Denmark, J.B.Winsløwvej 9, DK-5000 Odense, Denmark

**Keywords:** Cardiovascular disease, Prevention, Survey, Lifestyle, Barriers

## Abstract

**Background:**

Elimination of modifiable risk factors including unhealthy lifestyle has the potential for prevention of 80% of cardiovascular disease cases. The present study focuses on disclosing barriers for maintaining specific lifestyle changes by exploring associations between perceiving these barriers and various sociodemographic and health-related characteristics.

**Methods:**

Data were collected through a web-based questionnaire survey and included 962 respondents who initially accepted treatment for a hypothetical cardiovascular risk, and who subsequently stated that they preferred lifestyle changes to medication. Logistic regression was used to analyse associations between barriers to lifestyle changes and relevant covariates.

**Results:**

A total of 45% of respondents were identified with at least one barrier to introducing 30 min extra exercise daily, 30% of respondents reported at least one barrier to dietary change, and among smokers at least one barrier to smoking cessation was reported by 62% of the respondents. The perception of specific barriers to lifestyle change depended on sociodemographic and health-related characteristics.

**Conclusion:**

We observed a considerable heterogeneity between different social groups in the population regarding a number of barriers to lifestyle change. Our study demonstrates that social inequality exists in the ability to take appropriate preventive measures through lifestyle changes to stay healthy. This finding underlines the challenge of social inequality even in populations with equal and cost-free access to health care. Our study suggests supplementing traditional public campaigns to counter cardiovascular disease by using individualized and targeted initiatives.

## Background

The prevalence of cardiovascular mortality in Denmark and comparable countries has decreased during recent decades, but cardiovascular disease (CVD) remains a dominant cause of morbidity and mortality in many countries [1, 2]. Elimination of modifiable risk factors including unhealthy lifestyle allows for prevention of 80% of CVD cases [3], and individuals with desirable lifestyle factors (not smoking, physically active, healthy diet, BMI < 25) are expected to have a 67–72% lower risk of developing heart failure [4], However, actual implementation and maintenance of preventive measures, e.g. lifestyle change, has proven difficult [5–7]. Experiencing barriers to lifestyle change has been shown to prevent successful change of low exercise levels, unhealthy nutrition and/or smoking status among at-risk groups as well as in general populations [8–13].

Inability to maintain changes may be explained by limited structural resources (e.g. available time, or lacking financial resources, and influence by partner) or more personal challenges (e.g. habits, taste, and previous experience) [14]. Which types of barriers one perceives may, however, differ across population groups. In particular socio-economic factors might make a difference, but few studies have as yet systematically compared barrier perceptions across socio-economic strata. If specific barriers to lifestyle change can be identified and linked to specific population groups, it may be beneficial to approach lifestyle changes through targeted or individually tailored messages boosting self-efficacy and coping abilities, rather than using traditional and more generalized risk messages addressing mainly negative consequences of given lifestyles.

A recent survey of 40–60 year old Danes demonstrated that the vast majority of the study population expressed a preference for lifestyle changes instead of lifelong medication to prevent a hypothetical heart disease [15]. At the same time the respondents expressed doubt that they would be able to maintain the changed lifestyle for an extended period of time [16]. The present article is based on the same study population but focuses on disclosing group differences in perceived difficulties for maintaining specific lifestyle changes including exercise, diet, and smoking. The study explores whether perception of barriers commonly identified in the literature differs between socio-demographic groups and groups with different health status, different weight and different levels of physical activity.

In particular, the present study aims at investigating whether middle-aged Danes from different socio-economic backgrounds and with different health status vary in their perception of

1. Inexperience, time and cost as barriers to engaging in exercise
2. Taste, time and cost as barriers to eating low-fat food
3. Previous unsuccessful experience or lack of support from co-habitant(s) as barriers to stopping smoking

## Methods

### Sample and setting

A representative sample of 40–60 year old persons was invited to participate. This age group was considered to represent the most likely first time users of preventive therapy against CVD. The respondents were presented with a hypothetical scenario asking them to imagine that they were at increased risk of heart disease. Subsequently they were offered a hypothetical preventive medical intervention targeted at reducing the risk of heart disease. No medication name was mentioned. Afterwards, subjects were provided with the choice to either accept or reject this medication and further to indicate their preference for lifestyle changes versus medication. Lifestyle changes were framed as having the same benefit on CVD risk as medication. Eventually, respondents were asked about perceived barriers to maintaining specified lifestyle changes for 1 year (Table 1).

**Table 1:** Question presented to all respondents who had accepted treatment in the first place, but preferred lifestyle changes (another ½ hour daily exercise + low fat diet + no smoking) to medication

Which (lifestyle change) do you think will be hardest to maintain? (multiple answers are allowed)
○ Daily exercise for another ½ hour – I am not used to doing physical exercise ○ Daily exercise for another ½ hour – Work and children makes it hard to find the time ○ Daily exercise for another ½ hour – It is expensive to go to a fitness center ○ Low fat diet – It takes longer to cook compared to the usual cooking ○ Low fat diet – I don’t like low fat food ○ Low fat diet – many vegetables and fish are more expensive than the usual food ○ Stop smoking – I have tried several times ○ Stop smoking – I don’t think I can stop smoking when my cohabitant continues smoking ○ Other, please specify

Data were collected through a web-based questionnaire in March 2012 using TNS Gallup’s web-panel GallupForum (a panel consisting of 40.000+ Danish speaking persons >14 years of age). A random sample of 3928 panel members aged 40–60 years received an invitation by e-mail to visit a website, 2346 (60%) accessed the website, and 2099 (91%) answered the questionnaire. The full sample (18 groups) included variations over the size of risk reduction as well as delay in time before treatment benefit. The present paper uses a sub-sample (9 groups) consisting of those respondents who were presented with an immediate treatment benefit as an absolute risk reduction of 10% to 5% (*n* = 1069 out of 2099), who subsequently stated that they preferred lifestyle changes (½ hour daily exercise + low fat diet + no smoking) to medication (*n* = 962 out of 1069).

According to the Act on a Biomedical Research Ethics Committee System in Denmark, the project was not a biomedical research project and therefore did not need the ethic committee’s approval. All respondents were anonymous.

### Questionnaire

The questionnaire described a risk scenario together with 12 questions concerning health, such as self-rated health status, experience with heart disease (own/family), smoking, physical activity and willingness to accept treatment under various conditions, plus a number of questions on sociodemographic characteristics (gender, age, highest educational attainment, household income). The questionnaire has been used in previous studies [15, 16] except for questions on barriers. Based on the reviewed literature, three types of lifestyle change were selected together with two-three specific potential barriers to maintaining each of these changes for a year. The respondents were asked to state whether each of the barriers applied to them (Table 1). In addition, a free text option was provided to report further barriers. Prior to being presented to the web-panel, the entire questionnaire was evaluated regarding comprehensibility, relevance, acceptability and feasibility, and pilot tested by TNS Gallup. All respondents received a lottery ticket with the chance of winning goods worth 50 USD.

### Statistical analyses

Univariate and multivariable logistic regression was used to analyse associations between each predefined barrier to lifestyle changes and a number of covariates (gender, age, self-rated health status, body mass index (BMI), physical activity, experience with heart disease, education, work status, and household income). Tests for trends were conducted when appropriate, i.e. for continuous and ordinal variables. Only results from the multiple analyses, adjusted for gender and age as potential confounders, are presented in the paper. All analyses were conducted using Stata Release 11 (StataCorp, College Station, TX, USA).

## Results

Socio-economic characteristics of the 962 respondents who accepted the intervention and preferred life style changes are listed in Table 2. This subsample did not differ from the entire group of respondents (*n* = 2099) based on gender, age distribution, health status, household income, educational attainment, or occupational status. Moreover, for the age strata used, our sample of respondents was representative of the Danish population in terms of household income, educational attainment, and occupational status (data not shown).

**Table 2:** Sample characteristics

	Participants
Total, n	962
Gender, n (%)	
female	480 (49.9)
male	482 (50.1)
Age, mean (years)	50.6
Age groups, n (%)	
40–44	170 (17.7)
45–49	253 (26.3)
50–54	240 (24.9)
55–60	299 (31.1)
Health status, n (%)	
good/very good	620 (64.5)
fair	280 (29.1)
poor/very poor	61 (6.3)
BMI (kg/m^2^), n(%)	
< 25	370 (40.4)
25–29	352 (38.5)
+ 30	193 (21.1)
Physical activity^a^, n(%)	
low	284 (29.5)
high	678 (70.5)
Household income, n (%)	
low (< 80,000 USD)	286 (33.0)
medium	334 (38.5)
high (> 130,000 USD)	247 (28.5)
Educational attainment, n(%)	
low (<12 years schooling)	213 (22.3)
medium	645 (67.5)
high (university degree)	97 (10.2)
In work force, n(%)	
No	115 (12.0)
Yes	847 (88.0)

^a^Physical activity categorized as High: ‘daily’ or ‘several times a week’; Low: ‘never or ‘once a week or less’

### Barrier to life style change: increased daily exercise for 30 min

**Fig. 1:**
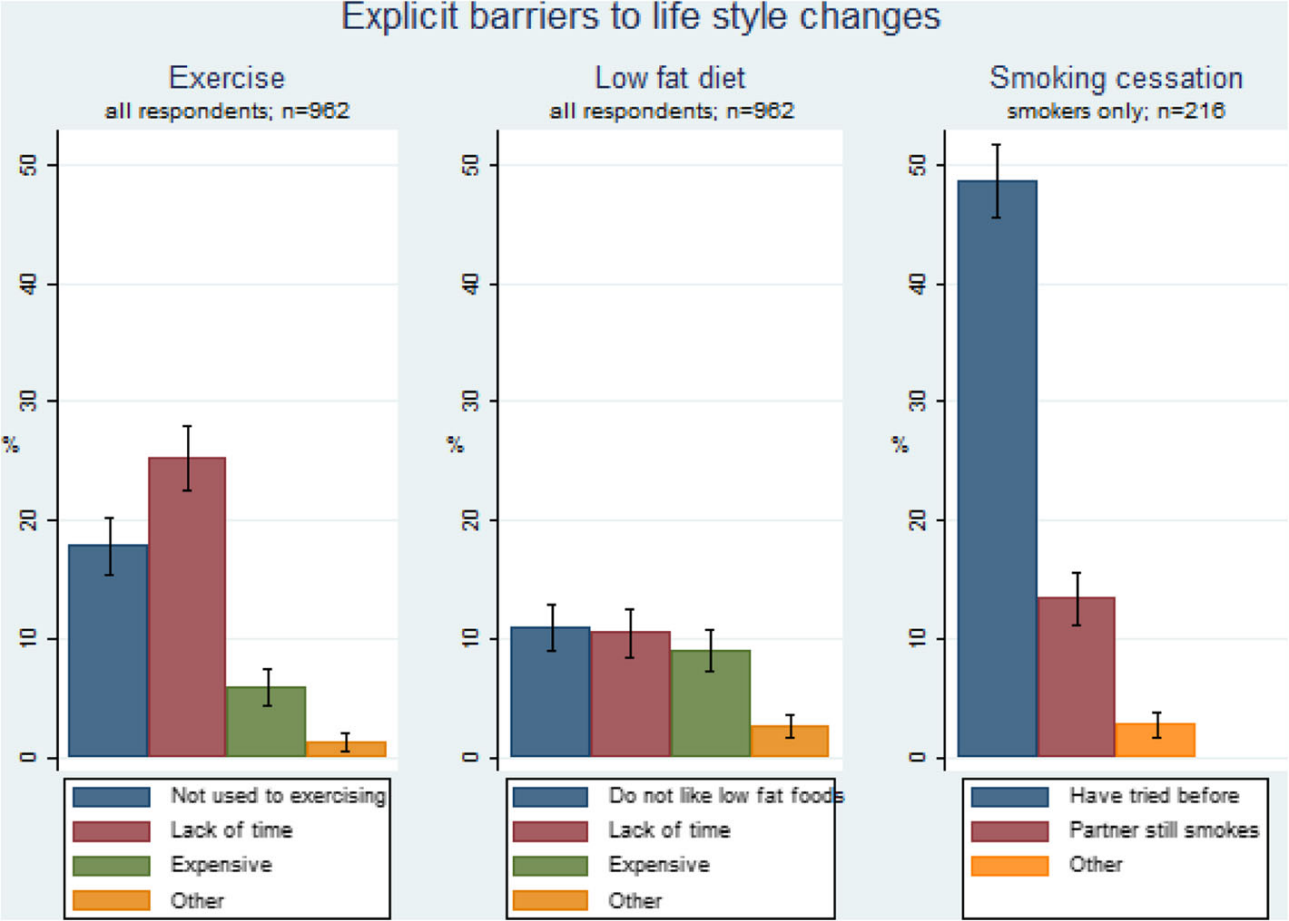
Explicit barriers to lifestyle changes (% of respondents)

A total of 45% of respondents indicated at least one of the three predefined barriers to introducing 30 min extra exercise daily. Lack of time for exercise was the most frequently reported barrier followed by not being used to do exercise and the cost of participating in organized exercise activities (Fig. 1).

The younger the respondents and the higher the income and level of educational attainment, the more often lack of time was reported as a barrier to ½ hour extra daily exercise (Table 3). Respondents in the work force significantly more often saw time as a barrier to exercise than those not working (OR = 5.70, *p* < 0.001).

**Table 3:** Associations between specific barriers for physical exercise and covariates among respondents who indicated a preference for lifestyle changes

		Total sample N	Not used to exercising n (%)^a^	Lack of time for exercising n (%)^a^	Too expensive to exercise n (%)^a^
Total		962	172 (17.9)	243 (25.3)	57 (5.9)
Gender	Male	482	93 (19.3) *p* = 0.309	134 (27.8) *p* = 0.045	29 (6.0) *p* = 0.993
	Female	480	79 (16.5)	109 (22.7)	28 (5.8)
Age group	40–44	170	22 (12.9) *p* = 0.075^*^	61 (35.9) p < 0.001^*^	8 (4.7) *p* = 0.144
	45–49	253	49 (19.4)	73 (28.9)	18 (7.1)
	50–54	240	37 (15.4)	58 (24.2)	8 (3.3)
	55–60	299	64 (21.4)	51 (17.1)	23 (7.7)
Health status	Good/very good	620	78 (12.6) p < 0.001^*^	162 (26.1) *p* = 0.251	30 (4.8) *p* = 0.129
	Fair	280	75 (26.8)	71 (25.4)	23 (8.2)
	Poor/very poor	61	19 (31.1)	10 (16.4)	4 (6.6)
BMI	<25	370	47 (12.7) p < 0.001^*^	100 (27.0) *p* = 0.611	9 (2.4) p < 0.001^*^
	25–29	352	60 (17.0)	91 (25.9)	28 (8.0)
	+30	193	55 (28.5)	47 (24.4)	20 (10.4)
Physical activity	Low	284	126 (44.4) p < 0.001	98 (34.5) *p* < 0.001	27 (9.5) *p* = 0.004
	High	678	46 (6.8)	145 (21.4)	30 (4.4)
Household income	Low	286	70 (24.5) *p* = 0.001^*^	54 (18.9) p = 0.001^*^	28 (9.8) p = 0.001^*^
	Medium	334	58 (17.4)	87 (26.0)	13 (3.9)
	High	247	31 (12.6)	80 (32.4)	8 (3.2)
Educational attainment	Low	213	45 (21.1) *p* = 0.026^*^	40 (18.8) p < 0.001^*^	13 (6.1) *p* = 0.159^*^
	Medium	645	117 (18.1)	160 (24.8)	42 (6.5)
	High	97	10 (10.3)	42 (43.3)	1 (1.0)
In work force	No	115	28 (24.3) *p* = 0.066	7 (6.1) p < 0.001	12 (10.4) *p* = 0.050
	Yes	847	144 (17.0)	236 (27.9)	45 (5.3)

All p-values are from multiple logistic regression analyses adjusted for gender and age

^*^- p-value for trend

^a^ Numbers (n) and percentages (%) correspond to the number of patients and proportions (in percent) of patients in the row-category who experienced the given barrier. Note that not all participants experienced a barrier within the theme, and that participants were allowed to select more than one barrier within the theme

Respondents already reporting high levels of physical activity less often saw lack of time for another 30 min of daily exercise as a barrier to maintaining a changed lifestyle (OR = 0.52, p < 0.001). We did not observe a significant association between lack of time for exercise and body mass index (BMI).

The lower the income the more frequently respondents perceived not being accustomed to exercising as a barrier. This was also true for those with lower levels of physical activity, with higher BMI and lower self-reported health status. Further, household income and self-reported level of present physical activity were negatively associated with the statement that it was too expensive to go to a fitness center (Table 3) while BMI was positively associated with perceiving cost of exercise as a barrier to maintaining lifestyle changes.

### Barriers to life style change: low fat diet

Some 30% of respondents reported at least one of the three predefined barriers to dietary change. Among these respondents a similar proportion stated barriers relating to not liking low fat food, low fat food taking longer to prepare, and low fat food being too expensive (Fig. 1). Males and individuals with lower self-reported health were more prone to stating that dislike of low fat food was a barrier to lifestyle change (Table 4). Further, the higher the BMI the higher the proportion of respondents reporting increased time demand as a barrier (Table 4).

**Table 4:** Associations between specific barriers for low fat diet and covariates among respondents who indicated a preference for lifestyle changes

		Total sample N	Do not like low fat foods n (%)^a^	Lack of time for cooking n (%)^a^	Low fat foods are too expensive n (%)^a^
Total		962	106 (11.0)	101 (10.5)	87 (9.0)
Gender	Male	482	83 (17.2) p < 0.001	54 (11.2) *p* = 0.428	47 (9.8) *p* = 0.422
	Female	480	23 (4.8)	47 (9.8)	40 (8.3)
Age group	40–44	170	17 (10.0) *p* = 0.875^*^	17 (10.0) *p* = 0.366^*^	18 (10.6) *p* = 0.044
	45–49	253	31 (12.3)	30 (11.9)	30 (11.9)
	50–54	240	25 (10.4)	30 (12.5)	19 (7.9)
	55–60	299	33 (11.0)	24 (8.0)	20 (6.7)
Health status	Good/very good	620	55 (8.9) *p* = 0.002^*^	51 (8.2) *p* = 0.008^*^	48 (7.7) *p* = 0.042^*^
	Fair	280	38 (13.6)	42 (15.0)	30 (10.7)
	Poor/very poor	61	13 (21.3)	8 (13.1)	9 (14.8)
BMI	<25	370	35 (9.5) *p* = 0.034	26 (7.0) p = 0.001^*^	17 (4.6) p < 0.001^*^
	25–29	352	37 (10.5)	37 (10.5)	37 (8.0)
	+30	193	32 (16.6)	32 (16.6)	28 (14.5)
Physical activity	Low	284	34 (12.0) *p* = 0.636	32 (11.3) *p* = 0.626	21 (7.4) *p* = 0.231
	High	678	72 (10.6)	69 (10.2)	66 (9.7)
Household income	Low	286	36 (12.6) *p* = 0.078^*^	21 (7.3) *p* = 0.154^*^	35 (12.2) *p* = 0.012^*^
	Medium	334	42 (12.6)	42 (12.6)	33 (9.9)
	High	247	21 (8.5)	28 (11.3)	15 (6.1)
Educational attainment	Low	213	24 (11.3) p = 0.428^*^	30 (14.1) *p* = 0.115^*^	28 (13.1) *p* = 0.005^*^
	Medium	645	71 (11.0)	63 (9.8)	57 (8.8)
	High	97	11 (11.3)	8 (8.2)	2 (2.1)
In work force	No	115	10 (8.7) *p* = 0.761	10 (8.7) *p* = 0.639	19 (16.5) p = 0.001
	Yes	847	96 (11.3)	91 (10.7)	68 (8.0)

All p-values are from multiple logistic regression analyses adjusted for gender and age

^*^- p-value for trend

^a^Numbers (n) and percentages (%) correspond to the number of patients and proportions (in percent) of patients in the row-category who experienced the given barrier. Note that not all participants experienced a barrier within the theme, and that participants were allowed to select more than one barrier within the theme

We also observed that being out of the workforce and high BMI were predictors of reporting monetary costs as a barrier to dietary change.

### Barriers to life-style change: smoking cessation

Among the 216 smokers, at least one of the two predefined barriers to smoking cessation was reported as a barrier to the required lifestyle change by 62%, most of who stated that they had previously unsuccessfully tried to stop smoking (49%), whereas ‘partner still smokes’ was stated as a barrier by 13% of the smokers. No significant associations were found between any of the covariates and the two barriers related to smoking cessation (Table 5).

**Table 5:** Associations between specific barriers to smoking cessation and covariates among respondents who indicated a preference for lifestyle changes

		Total sample of smokers N	Have tried before n (%)	Partner still smokes n (%)
Total		216	105 (48.6)	29 (13.4)
Gender	Male	115	56 (48.7) *p* = 0.992	13 (11.3) *p* = 0.318
	Female	101	49 (48.5)	16 (15.8)
Age group	40–44	47	28 (59.6) *p* = 0.051	5 (10.6) *p* = 0.360
	45–49	53	26 (49.1)	7 (13.2)
	50–54	53	26 (49.1)	6 (11.3)
	55–60	63	25 (39.7)	11 (17.5)
Health status	Good/very good	115	60 (52.2) *p* = 0.560^*^	11 (9.6) *p* = 0.183^*^
	Fair	83	34 (41.0)	16 (19.3)
	Poor/very poor	18	11 (61.1)	2 (11.1)
BMI	<25	89	42 (47.2) *p* = 0.949	13 (14.6) *p* = 0.744^*^
	25–29	72	38 (52.1)	9 (12.3)
	+30	45	20 (44.4)	6 (13.3)
Physical activity	Low	84	41 (48.8) *p* = 0.835	12 (14.3) *p* = 0.839
	High	132	64 (48.5)	17 (12.9)
Household income	Low	82	37 (45.1) *p* = 0.892^*^	12 (14.6) *p* = 0.904^*^
	Medium	66	34 (51.5)	8 (12.1)
	High	44	20 (45.5)	7 (15.9)
Educational attainment	Low	65	38 (58.5) p = 0.154^*^	9 (13.8) *p* = 0.623^*^
	Medium	130	58 (44.6)	18 (13.8)
	High	20	9 (45.0)	2 (10.0)
In work force	No	35	17 (48.6) *p* = 0.732	2 (5.7) *p* = 0.105
	Yes	181	88 (48.6)	27 (14.9)

All p-values are from multiple logistic regression analyses adjusted for gender and age

^*^- p-value for trend

^a^Numbers (n) and percentages (%) correspond to the number of patients and proportions (in percent) of patients in the row-category who experienced the given barrier. Note that not all participants experienced a barrier within the theme, and that participants were allowed to select more than one barrier within the theme

### Barriers to life-style change: free text option

Analysis of the free text option in the questionnaire did not reveal any major barriers not covered by those themes already included.

## Discussion

The present study indicates that the part of the population at increased risk for CVD (either BMI > 25, or those physically inactive) more often report the specified barriers to lifestyle change than population segments at lower risk for CVD. Even though creating time for physical activity/exercise or ensuring room in the budget to buy healthier food may be seen as a prioritization issue, our study clearly demonstrates that structural factors, such as low income and being out of the work force are associated with stating that low fat foods are too expensive and that it is too costly to use fitness centers to support lifestyle changes. Similarly, previous studies have shown that being unemployed or having low income and low socioeconomic position is associated with lower participation in and higher drop-out from cardiac rehabilitation programs [17, 18]. Thus, social inequality exists not only in the delivery and offering of health care services, but also in the ability to take appropriate preventive measures through lifestyle changes to stay healthy. This finding underlines the challenge of social inequality even in an otherwise fairly homogeneous Danish population with apparently equal and cost-free access to health care.

Among the barriers most commonly reported were those relating to cost and time. This may raise the question as to whether some respondents might claim these as barriers because they are aware that they have a health issue needing personal investment (time, money, convenience), but doubt their own ability or true intention to act on it [16]. This could be a kind of coping mechanism transforming the challenge of lifestyle change from being a personal issue to a more societal or structural issue related to costs and lack of time. Testing subgroup differences in perception of specified physical activity barriers, which had been identified as relevant in prior studies, may unintendedly have prompted some respondents to think only of fitness centers rather than of incorporating 30 min physical activity as a part of everyday life (walking or cycling to work) or without incurring much additional cost by jogging/running in the neighborhood).

Besides structural barriers, we considered more personal barriers related to habits and preferences. Not being used to exercise/physical activity, not liking low fat food, or for smokers having a partner who smokes were each regarded as a barrier to maintaining lifestyle change by at least 10% of the respondents. This is in accordance with the literature reporting that attitude towards and experience with physical activity/exercise, unwillingness to change diet, and family support are important determinants for maintaining initiated lifestyle changes [14, 17].

When perceived barriers are not equally distributed across socio-demographic strata, it underpins the need for a targeted or an individualized approach to CVD prevention through lifestyle changes. Further, it opens for a discussion on the dilemma between a societal wish to promote healthy lifestyles and the existence of population groups unwilling or unable to engage in smoking cessation or in losing weight despite being overweight or obese. In that way, our observations align with previous findings of a discrepancy between what people intend to do if at risk, and what individuals at risk actually do to prevent CVD.

The single most frequently reported barrier to lifestyle changes, stated by 25% of our respondents, was finding ½ an hour extra time to do daily exercise. Demographically these respondents were younger, in the work force, had higher household income and a higher educational attainment. The last three determinants were positively associated with lack of time as perceived barrier for physical exercise, but the associations with age were more complex. Thus, what characterizes this barrier to lifestyle change is diversity, which may also be part of the explanation why only some but not all previous studies have found lack of time or time stress to be a barrier to lifestyle changes [6, 14].

Unlike the barriers on diet and physical activity, we found no statistically significant sociodemographic/socioeconomic gradients for perceived barriers to smoking cessation. For educational attainment and health status this may be due to the reduced sample size.

In the present study, the respondents were presented with a limited number of suggested barriers to consider as well as a free text option. This may be seen as a limitation, since it might direct the respondent’s attention to the barriers suggested in the questionnaire which might lead to underestimation of the overall number of barriers perceived by people trying to change their lifestyle. However, the purpose of the present study was not to explore the overall number and range of possible barriers, but to test subgroup-specific differences in barriers which had been identified as the most relevant ones in the literature [14, 17, 19]. Further, examining the free text option in the questionnaire did not reveal any major topics not already covered by the themes. Nevertheless it is possible that the specification of only 2 to 3 specific options, because of cognitive availability, acquiescence bias or conformity processes, may have led to higher endorsement of these barriers at the expense of other, non-listed choices. We do multiple comparisons and therefore have an increased risk for type I errors. We have handled this by focusing on differences that are both relevant in size and having *p*-values below 0.005. Another important limitation may be that our respondents were asked to state their intentions and beliefs referring to a hypothetical risk situation, rather than to a real-life CVD risk. However, the subsample of our population reporting either to have a heart disease themselves or having family members with heart disease (in total 28% of our respondents) gave similar responses to barriers for lifestyle changes as the majority of our respondents with no prior experience with heart disease. As the present study population is extracted from a representative population sample based on criteria on willingness to change lifestyle and avoid medication, we cannot expect the chosen sample to be fully representative of the entire population. However, from previous data from Statistics Denmark we have previously reported that within the present age group the average household income is appr. 100,000 USD, 28% have less than 12 years schooling, and 7% have a University degree [20]. Thus, despite that our group is a subpopulation based on the above mentioned criteria; they appear fairly representative of the general population regarding these sociodemographic characteristics. Further strengths of the study were the solid sample size of 962 respondents, the high participation rate (91%) among those accessing the website for the study, that respondents were not informed about the specific risk scenarios before entering the survey, and that the questionnaire was thoroughly tested before being used.

An important observation from our study is the considerable heterogeneity between different social groups in the population regarding a number of barriers to lifestyle change, an issue, which has so far not been given sufficient attention. Thus, traditional generalized preventive efforts based on ‘one size fits all’ may not be optimal. A recent review concludes that prevention is cost-effective and should be delivered at the general population level by promoting healthy lifestyle behavior, and at the individual level by tackling unhealthy lifestyles (e.g. poor-quality diets, physical inactivity, smoking) [7]. This is in accordance with our study, suggesting to change or supplement traditional general public campaigns to counter CVD by using individualized and targeted initiatives directed at the groups at highest risk.

## Conclusion

We observed a considerable heterogeneity between different social groups in the population regarding a number of barriers to lifestyle change. Our study demonstrates that social inequality exists in the anticipated ability to take appropriate preventive measures through lifestyle changes to stay healthy. While our study is based on a hypothetical scenario and any conclusions are therefore tentative, the findings suggest that it may be worth wile to consider supplementing traditional public campaigns to counter cardiovascular disease, for instance by using individualized and targeted initiatives directed at the groups at highest risk.
